# A Cautionary Tale of Hypertrophic Cardiomyopathy—From “Benign” Left Ventricular Hypertrophy to Stroke, Atrial Fibrillation, and Molecular Genetic Diagnostics: A Case Report and Review of Literature

**DOI:** 10.3390/ijms25179385

**Published:** 2024-08-29

**Authors:** Dolina Gencheva, Petya Angelova, Kameliya Genova, Slavena Atemin, Mila Sleptsova, Tihomir Todorov, Fedya Nikolov, Donka Ruseva, Vanyo Mitev, Albena Todorova

**Affiliations:** 1 First Department of Internal Diseases, Medical University of Plovdiv, 4002 Plovdiv, Bulgaria; 2 Clinic of Cardiology, University Multi-Profile Hospital for Active Treatment “Sveti Georgi”, 4002 Plovdiv, Bulgaria; 3 Department of Medical Chemistry and Biochemistry, Medical University–Sofia, 1431 Sofia, Bulgaria; 4 Radiology Department, University Multi-Profile Hospital for Active Treatment and Emergency Medicine “N. I. Pirogov”, 1606 Sofia, Bulgaria; 5 Genetic Medico-Diagnostic Laboratory “Genica”, 1612 Sofia, Bulgaria; slavena_tsaneva@abv.bg (S.A.);; 6 Clinic of Cardiology, Hospital of Ministry of Transport, 4004 Plovdiv, Bulgaria

**Keywords:** genetic testing, hypertrophic cardiomyopathy, left ventricular hypertrophy, cardiac magnetic resonance, coronary slow-flow phenomenon, atrial fibrillation, whole-exome sequencing, *ACTC1*, *PLN*, *SCN5A*

## Abstract

This case report concerns a 48-year-old man with a history of ischemic stroke at the age of 41 who reported cardiac hypertrophy, registered in his twenties when explained by increased physical activity. Family history was positive for a mother with permanent atrial fibrillation from her mid-thirties. At the age of 44, he had a first episode of persistent atrial fibrillation, accompanied by left atrial thrombosis while on a direct oral anticoagulant. He presented at our clinic at the age of 45 with another episode of persistent atrial fibrillation and decompensated heart failure. Echocardiography revealed a dilated left atrium, reduced left ventricular ejection fraction, and an asymmetric left ventricular hypertrophy. Cardiac magnetic resonance was positive for a cardiomyopathy with diffuse fibrosis, while slow-flow phenomenon was present on coronary angiography. Genetic testing by whole-exome sequencing revealed three variants in the patient, c.309C > A, p.His103Gln in the *ACTC1* gene, c.116T > G, p.Leu39Ter in the *PLN* gene, and c.5827C > T, p.His1943Tyr in the *SCN5A* gene, the first two associated with hypertrophic cardiomyopathy and the latter possibly with familial atrial fibrillation. This case illustrates the need for advanced diagnostics in unexplained left ventricular hypertrophy, as hypertrophic cardiomyopathy is often overlooked, leading to potentially debilitating health consequences.

## 1. Introduction

Hypertrophic cardiomyopathy (HCM) [OMIM: 192600] is a common hereditary disease of the myocardium, characterized by a complex pathophysiology with variable clinical manifestations and disease course in individual patients [1]. Its estimated prevalence in the general population is 1:500 to 1:200 [2,3]. HCM is caused by mutations in at least 20 genes, encoding cardiac sarcomere proteins and sarcomere-associated proteins, while a genetic cause cannot be identified in 40% to ~65% of cases [4,5,6].

HCM is mainly inherited in an autosomal dominant pattern, but an autosomal recessive or X-linked inheritance is rarely possible [7]. Mutations in the *MYH7* and *MYBPC3* genes, encoding thick myofilament β-myosin heavy chain and myosin binding protein C, respectively, are most commonly found, in approximately 80% of patients with HCM with a positive genetic test result, followed by mutations in the troponin T (*TNNT2*), troponin I (*TNNI3*), and α-tropomyosin (*TPM1*) genes of thin myofilaments, which are identified in about 15% of patients [4,8]. Pathogenic mutations have also been reported in the genes for troponin C (*TNNC1*), regulatory myosin light chain (*MYL2*), essential myosin light chain (*MYL3*), α-actin (*ACTC1*), as well as LIM binding domain 3 (*LDB3*) in approximately 5% of HCM patients. Mutations in the genes *MYH6* (α-myosin heavy chain), *TTN* (titin), *ACTN2* (α-actinin 2), *MYOZ2* (myozenin 2), *CSRP3* (muscle LIM protein), *TCAP* (telethonin), *VCL* (vinculin), *ANKRD1* (ankyrin repeat domain 1), *FLNC* (filamin C), *MYPN* (myopalladin), *NEXN* (nexilin), and genes related to calcium metabolism, such as phospholamban (*PLN*), have been reported rarely, in less than 1% of positive patients. Two (homozygous, double, or compound heterozygous) or three pathogenic mutations in one or more genes have been identified in 5% and 0.8% of HCM patients, respectively [9,10,11].

In HCM, the occurring changes in the myocardium are not provoked by any abnormal hemodynamics; however, it is important to note that the coexistence of HCM and other conditions is not uncommon and might lead to misdiagnosis. The expression of the diverse genetic mutations in HCM leads to an age-dependent appearance of myocyte hypertrophy with significant enlargement of the myocyte diameter. The hypertrophy is often disproportionately asymmetric and predominantly involves the interventricular septum [12,13]. Diagnosis is typically suspected on standard echocardiography, but the presence of low-grade ventricular hypertrophy in the earlier stages or confusion with athlete’s heart in seemingly young and healthy individuals could result in underdetection [14,15,16]. In those situations, and in order to exclude phenocopies that can be due to storage diseases, more advanced methods are needed, such as cardiac magnetic resonance (CMR) and genetic testing. Both tests provide the clinician with more certainty for the establishment of the diagnosis, which has particularities in its follow-up and treatment, while molecular genetic testing has the added value of identifying relatives of the proband at risk [17].

This case report aims to illustrate that even though HCM is not an uncommon disease, its presence could be overlooked even in the era of modern medicine, leading to serious health-related consequences.

## 2. Detailed Case Description

The case report concerns a now 48-year-old man, in whom the diagnosis of HCM was not suspected or established before several significant complications of the condition had occurred. A summarized timeline of the more important events is presented in Table 1.

In his early twenties, the patient was reportedly told that he had “hypertrophy of the heart”, but the phenomenon was categorized as benign and associated with the strenuous physical effort during military service. No further follow-up was recommended. In 2016, at the age of 41, the patient suffered a right middle cerebral artery ischemic stroke. Doppler sonography of the carotid arteries and the vertebrobasilar system was described as within normal limits and no rhythm or conduction disturbances were noted during his monitoring. A chest X-ray revealed enlargement of the heart, which was not further investigated. The main risk factor for the stroke was deemed to be arterial hypertension that he had been having for a few years and was well controlled with Irbesartan 75 mg daily with no hypertensive crises detected at admission or during the hospital stay. The patient was left with a residual left-sided hemiparesis and a central-type lesion of the facial nerve, which improved significantly over time.

In July 2020, a first episode of persistent atrial fibrillation was detected and anticoagulant treatment with the direct oral anticoagulant (DOAC) Dabigatran 150 mg bid and Bisoprolol 2.5 mg bid was initiated. A month later, he visited a different cardiology clinic for a planned transesophageal echocardiography (TEE) and electrical cardioversion (ECV). The TEE, however, was positive for thrombosis in the left atrial appendage (LAA) and the DOAC was switched with the vitamin K antagonist (VKA) acenocoumarol. Two months later, the LAA was negative for thrombosis on TEE and sinus rhythm was restored at the same clinic. The ECG at admission was described as atrial fibrillation of 120 beats per minute (bpm) and right bundle branch block (RBBB). An enlarged left atrium (LA) with an anterior–posterior diameter of 49.5 mm and a normal left ventricular ejection fraction (LVEF) of 53% were reported from the transthoracic echocardiography. Left ventricular hypertrophy was noted with the septum, measuring 14 mm, and the posterior wall of the left ventricle (PWLV) 12.4 mm, leading to the conclusion that a symmetrical hypertrophy was present. The patient was discharged on acenocoumarol, bisoprolol 2.5 mg bid, and irbesartan 75 mg. Of note, the thyroid-stimulating hormone (TSH) was within normal limits during this hospital stay—2.127 mU/L.

Approximately 6 months later, in March 2021, the patient presented at our Cardiology Clinic with an episode of decompensated heart failure, New York Heart Association (NYHA) functional class III–IV, as well as another episode of persistent atrial fibrillation. The latter was registered by his general practitioner two months earlier and outpatient treatment with 400 mg amiodarone daily was initiated. The patient reported having pretibial edema and progressive dyspnea for the past two months despite taking 20 mg of torasemide. At admission, he was in a moderately impaired general condition, tachy-dyspneic and orthopneic. Lung auscultation was positive for moist rales bilaterally at the bases of the lungs. Heart auscultation did not reveal any pathological murmurs; heart sounds were dull, likely due to his hypersthenic composure; heart rate (HR) was arrhythmic with 90 bpm; and blood pressure (BP) was 100/60 mmHg. Hepatomegaly of about 4 cm below the costal arch was noted and there was massive symmetric bilateral pretibial pitting edema. The patient weighed 130 kg at the time, his height was 180 cm, body mass index (BMI) was 40.1 kg/m^2^, and body surface area (BSA) was 2.52 m^2^ by the Mosteller’s formula.

**Figure 1:**
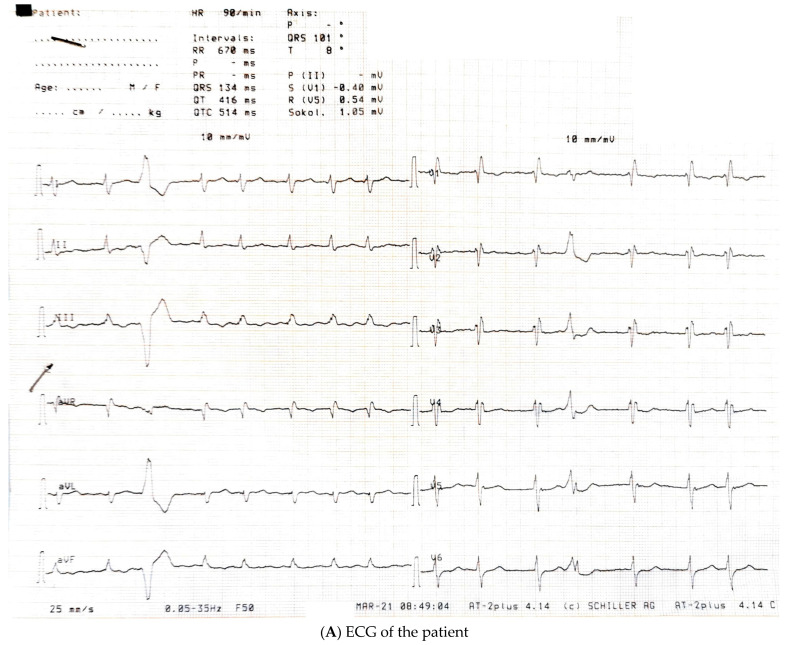

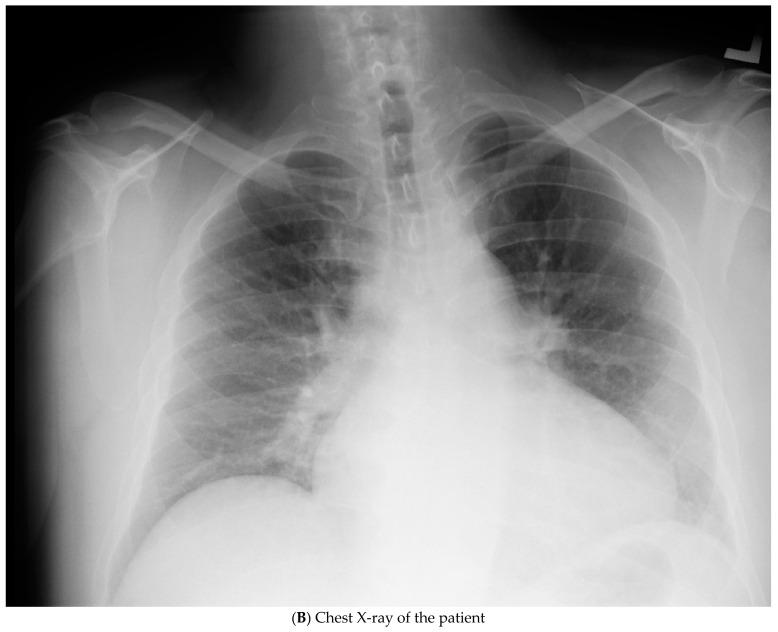
(**A**) Electrocardiogram (ECG) at admission showing atrial fibrillation with a frequency of about 90 beats per minute, right axis deviation, and a right bundle branch block with a QRS complex duration of 134 ms. A single ventricular extrasystole is noted. (**B**) Chest X-ray of the patient at admission showing cardiomegaly and pulmonary congestion.

The patient denied smoking, alcohol abuse, or use of illegal substances. His arterial hypertension was with maximum detected blood pressure values of 150/90 mmHg (grade I) and was well-controlled, with episodes of hypotension reported with the current treatment. Family history was positive for a mother with chronic atrial fibrillation from as early as her mid-thirties and a father who had arterial hypertension and died from an unrelated, non-cardiovascular disease. The ECG and chest X-ray at admission are presented on Figure 1.

Laboratory tests revealed no anemia, leukocytosis, or other abnormalities from the full blood count; creatinine, urea, potassium, sodium and chloride ions, aspartate transferase, alanine transaminase, total protein, albumin, fibrinogen, C-reactive protein, troponin I, and D-dimers were all within normal limits. The serum uric acid was elevated at 513.0 µmol/L and the international normalized ratio of the prothrombin time (INR) was 1.15, indicating a lack of adequate anticoagulation. Total cholesterol was 4.8 mmol/L; HDL cholesterol—1.2 mmol/L; triglycerides—1.8 mmol/L; and LDL cholesterol—2.8 mmol/L. Thyroid-stimulating hormone (TSH) was low at 0.048 mU/L, and free triiodothyronine (fT3) and free thyroxine (fT4) were elevated at 42.01 pmol/L and 8.88 pmol/L, respectively indicating hyperthyroidism.

### 2.1. Echocardiographic Study

**Figure 2:**
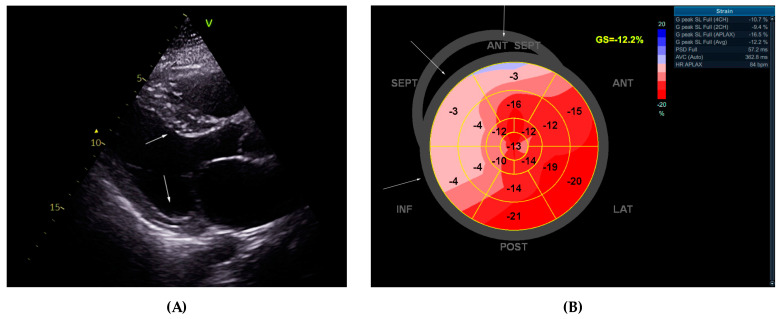
Transthoracic echocardiography findings: (**A**) Parasternal long axis view showing hypertrophy of the septum, visibly more pronounced, compared to the posterior wall of the left ventricle (16 mm vs. 8 mm) with an estimated asymmetricity index of 2. (**B**) Speckle tracking global longitudinal strain of the left ventricle (LV GLS) showing an average value of 12.2% with regionally reduced strain values at the basal and mid-portions of the septum, the inferior wall, and the anteroseptal wall. Peak systolic dispersion (PSD) was 57.2 ms. Abbreviations of the left ventricular segments from (**B**) SEPT—septum; ANT SEPT—anterior septal wall; ANT—anterior wall; LAT—lateral wall; POST—posterior wall; INF—inferior wall.

Transthoracic echocardiography was performed, and the findings differed significantly from those previously described, possibly due to obesity-related suboptimal imaging. Details are given in Table 2 and Figure 2. While atrial dilation was indeed present with a left atrium volume index (LAVI) 52 mL/m^2^, the left ventricular hypertrophy was asymmetrical with the basal septum being 16–17 mm and the PWLV 8 mm (asymmetricity index of 2). There was no intraventricular gradient or left ventricular outflow tract obstruction. The LVEF (Simpson’s biplane method of discs) was reduced—about 35–40%. Diffuse hypokinesia of the septum was noted during the exam, which was confirmed by speckle tracking global longitudinal strain in the left ventricle (LV GLS). The latter showed significantly impaired regional kinetics encompassing the basal and mid-portions of the septum, the inferior wall, and the anteroseptal wall. Diastolic dysfunction of the left ventricle was also present. The right ventricular systolic function was borderline low with normal thickness of the free wall. Mild mitral and tricuspid insufficiencies were present. A suspicion was raised for the presence of a cardiomyopathy. The patient was scheduled for cardiac magnetic resonance (CMR). The alpha-galactosidase was 19.7 µmol/L/h (reference range ≥ 15.3 µmol/L/h, fluorimetry method), ruling out Anderson–Fabry disease.

TEE was not performed at the time due to the patient not being in the therapeutic interval for VKA treatment; a stricter control of the INR was recommended, and TEE was planned a month later. The CHA2DS2-VASc score for the patient was 4 points (past ischemic stroke, low LVEF, and arterial hypertension), with a HAS-BLED score of 1 (previous stroke). Therapy at discharge (Table 1) was consistent with the available European Society of Cardiology guidelines for the treatment of heart failure with reduced ejection fraction [18] and an SGLT2 inhibitor was also included [19]. Regarding the thyroid dysfunction, thyroglobulin antibodies and thyroid peroxidase antibodies were below the detectable limit and there were no structural abnormalities of the thyroid gland. Treatment with prednisolone was initiated and future use of amiodarone was strongly discouraged as a likely cause for the hyperthyroidism. Euthyroid state was achieved after a few months and persisted at follow-up.

### 2.2. Cardiac Magnetic Resonance (CMR)

**Figure 3:**
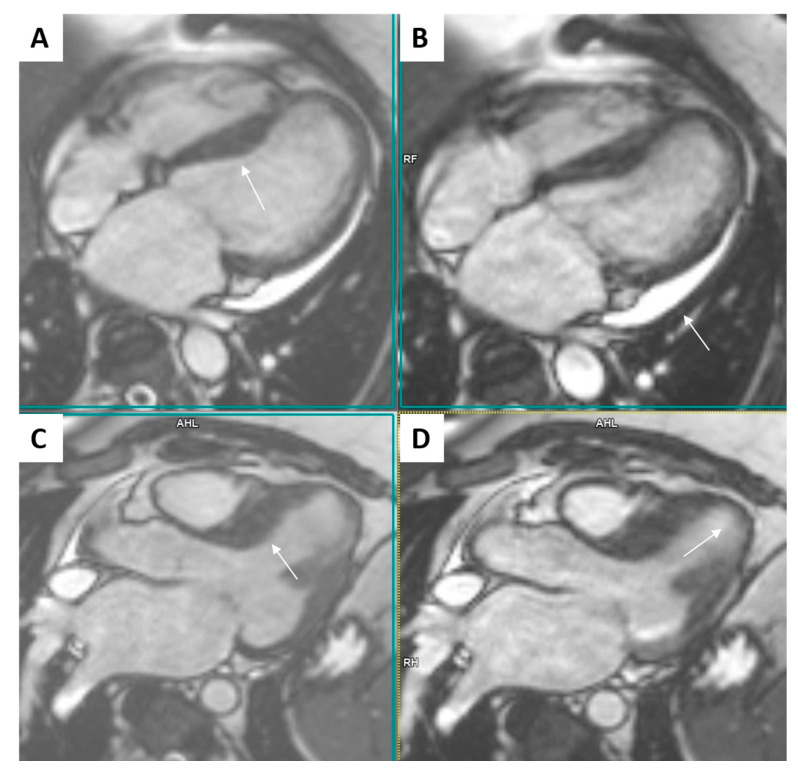
Cardiac magnetic resonance findings. Four chamber (first line) and three chamber (second line) SSFP CINE images in the end diastole (**A**,**C**) and end systole (**B**,**D**). Mildly thickened middle and partially basal septum (up to 16 mm, arrows in (**A**,**C**)) with the borderline thinned myocardium along the apical free wall and at the apex (arrow in (**D**)). Pericardial effusion along the free wall of the left ventricle (Arrow in (**B**)).

**Figure 4:**
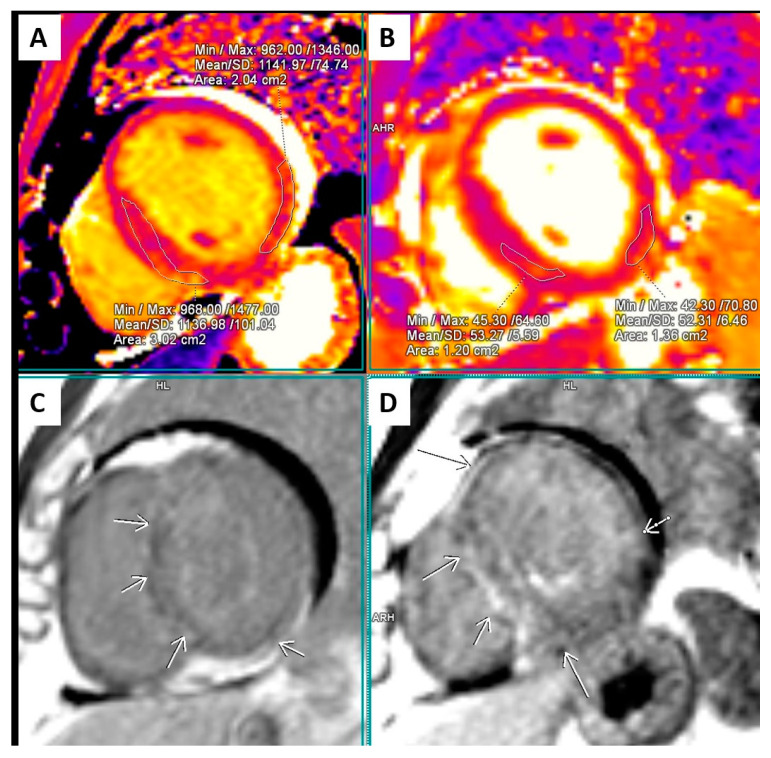
Cardiac magnetic resonance findings. Short-axis T1 (**A**) and T2 (**B**) mapping and LGE images (**C**,**D**). Variably prolonged T1 relaxation time (mean 1140 ms, (**A**)) and T2 relaxation time (mean 53 ms, (**B**)) along the septum and the free wall of the left ventricle. Patchy and linear LGE in depth along the basal and middle septum and along the lateral and lower-lateral wall of the left ventricle. Limited subepicardial LGEI was also detected.

The CMR study (Figure 3 and Figure 4) confirmed the asymmetric hypertrophy of the left ventricle with mid and basal septum thickness 16 mm, while the rest of the left ventricular walls were 3–6 mm thick. The LVEF was 34.8% with an end-diastolic volume of the LV 184 mL (indexed to BSA 75 mL/m^2^), an end-systolic volume 120 mL (indexed to BSA 48.9 mL/m^2^), indexed stroke volume of 26.1 mL/m^2^, cardiac index 2.27 L/min/m^2^, and myocardial mass index 76.7 g/m^2^. Profound hypokinesia of the basal and apical septum, the apical anterior wall, and the basal inferior wall as well as moderate hypokinesia of the rest of the left ventricular segments was confirmed. The right ventricle was not dilated, with an end-diastolic volume of 104 mL (indexed 42.5 mL/m^2^) and an end-systolic volume of 63.9 mL (indexed 26 mL/m^2^), but its ejection fraction was reduced to 38.5% with diffuse moderate hypokinesia. The right ventricular free wall thickness was in the reference range. The left atrium was significantly enlarged (67.43 mL/m^2^), while the right atrium was within reference range. No significant valvular lesions were detected.

The T2-weighted turbo inversion recovery magnitude (T2 TIRM) images showed intense strong signals apically of the left ventricle, as well as patchy low-intensity signals in the mid-portion septum and in certain zones of the basal portion of the left ventricular lateral wall. The T2 relaxation time in those areas was significantly prolonged, varying widely—between 58 and 88 ms—confirming increased myocardial water content. Intense intramural late gadolinium enhancement (LGE) was present in the basal, middle, and apical portion of the septum and was more pronounced in the hypertrophied regions. Linear and patchy LGE was present in all segments of the LV intramurally, but at the apex and the apical lateral wall, it also had a subendocardial localization, engaging more than 50% of the myocardial thickness. LGE was present in the free wall of the RV as well. The T1 relaxation time in those zones was significantly prolonged—between 1250 ms and 1350 ms. The presence of both LGE and prominently prolonged T1 relaxation time indicated enlargement of the myocardial interstitial space.

The conclusion of the CMR reading was for a cardiomyopathy with moderate septal hypertrophy and a diffuse myocardial involvement, possibly an infiltrative cardiomyopathy; however, reverse remodeling due to hypertrophic cardiomyopathy could not be ruled out. The presence of limited segmental disturbances in kinetics with subendocardial LGE raised the suspicion of a concomitant coronary artery disease, and selective coronary angiography was advised. Genetic testing was also recommended.

### 2.3. Follow-Up

**Figure 5:**
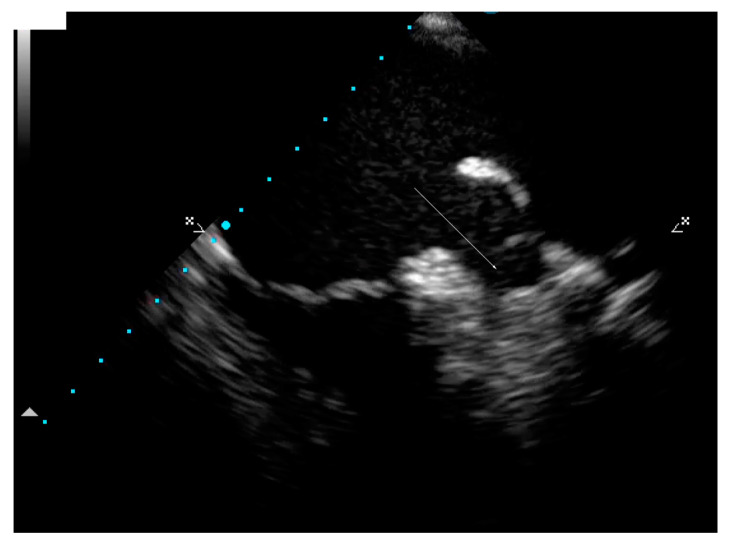
Transesophageal echocardiography showing thrombosis of the left atrial appendage (arrow).

In April 2021, TEE was performed, while in a therapeutic anticoagulant interval (INR 2.22), but nonetheless, it revealed the presence of a LAA thrombosis (Figure 5). A rate control strategy was adopted. Coronary angiography was performed due to the CMR-detected subendocardial involvement and while negative for coronary atherosclerotic disease, it revealed a slow-flow phenomenon in all coronary arteries. The patient was consulted with a genetics specialist and agreed to undergoing clinical genetic testing.

The next visit of the patient was in October 2021. INR at that visit was again not on target, 0.94, due to irregular follow-ups and lack of adherence. Overall, some positive development was noticed—his ejection fraction has improved with treatment and was around 50%. The patient reported mild to no functional class limitation. Torasemide was reduced to 10 mg and later on to 5 mg daily. Holter ECG did not reveal any significant ventricular ectopic activity, and the ventricular rate was well controlled with a mean HR of 82.

### 2.4. Molecular-Genetic Analysis

**Figure 6:**
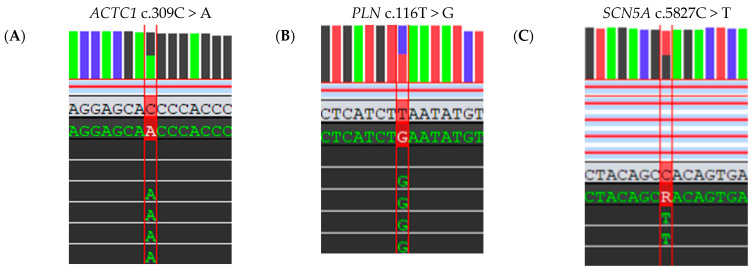
Molecular genetic analysis by whole-exome sequencing and a targeted panel of 242 cardiomyopathy-associated genes revealed 3 variants in the proband: c.309C > A, p.His103Gln in the *ACTC1* gene (**A**) and c.116T > G, p.Leu39Ter in the *PLN* gene (**B**), as well as c.5827C > T, p.His1943Tyr in the *SCN5A* gene (**C**). The *ACTC1*, *PLN*, and *SCN5A* gene variants wre reported using RefSeq NM_005159.5, NM_002667.5, and NM_001160161.2, respectively. (**A**) ACMG/AMP classification: likely pathogenic (categories: PP3, PM5, PM2, PP2). (**B**) ACMG/AMP classification: pathogenic (categories: PVS1, PP5, PM2). (**C**) ACMG/AMP classification: variant of uncertain significance (category: PM2).

A blood sample was collected from the patient and DNA was extracted using the salting-out method. Molecular genetic analysis by whole-exome sequencing (WES) and a targeted panel of 242 cardiomyopathy-associated genes revealed the following three variants: c.309C > A (p.His103Gln) in the *ACTC1* gene, c.116T > G, p.Leu39Ter in the *PLN* gene, as well as c.5827C > T, p.His1943Tyr in the *SCN5A* gene (Figure 6).

Pathogenic heterozygous variants in the *ACTC1* gene and the *PLN* gene have been reported to cause hypertrophic cardiomyopathy 11 [OMIM: 612098] and 18 [OMIM: 613874], respectively. Variants p.His103Gln in the *ACTC1* gene and p.Leu39Ter in the *PLN* gene have both been previously described in ClinVar and literature databases. The variant p.His103Gln in the *ACTC1* gene has not been found in the control populations of the gnomAD v2.1.1 project and variant p.Leu39Ter in the *PLN* gene has an allele frequency of 0.00001592; homozygotes have not been reported. In silico analysis of the variant p.His103Gln in the *ACTC1* gene by PolyPhen-2, SIFT, and MutationTaster predicted it as damaging, with position 103 being highly conserved and histidine and glutamine having a small physicochemical difference. MutationTaster predicted variant p.Leu39Ter in the *PLN* gene as damaging in line with the expected loss of protein function due to a premature termination codon at position 39. According to the ACMG/AMP criteria, variants p.His103Gln in the *ACTC1* gene and p.Leu39Ter in the *PLN* gene may be classified as likely pathogenic and pathogenic, respectively.

Moreover, pathogenic heterozygous and homozygous variants may cause *SCN5A*-related conditions, including familial atrial fibrillation 10 [OMIM: 614022]. The variant p.His1943Tyr in the *SCN5A* gene has not been previously reported in ClinVar or the public literature or identified in the control populations of the gnomAD v2.1.1 project. In silico predictors show conflicting results of pathogenicity and there is moderate physicochemical difference between histidine and tyrosine (PolyPhen-2—benign; SIFT—damaging; MutationTaster—polymorphism). Therefore, the variant p.His1943Tyr in the *SCN5A* gene may be classified as a variant of uncertain significance, according to ACMG/AMP criteria.

In conclusion, WES led to the detection of two heterozygous variants in the *ACTC1* gene and in the *PLN* gene, which represent the probable cause of hypertrophic cardiomyopathy in the patient. Additionally, a heterozygous variant of uncertain clinical significance in the *SCN5A* gene, associated with familial atrial fibrillation, was identified.

### 2.5. Diagnosis and Further Follow-Up

Given the molecular genetic testing, the CMR findings, and the clinical presentation, our final diagnosis for the patient was HCM. The calculated risk of sudden cardiac death was 2.39% (calculated at https://professional.heart.org/en/guidelines-and-statements/hcm-risk-calculator (accessed on 15 July 2024)), which did not necessitate the implantation of an implantable cardioverter–defibrillator device (ICD) for primary prevention of sudden cardiac death [14]. Newer reports, related to the presence of LGE and its prognostic value, however, might soon change the perspective on ICD implantation [20].

The patient declined referral for pulmonary vein isolation (PVI) for the atrial fibrillation should the LAA be negative for thrombosis. In the following years, he remained in an NYHA class I of HF, did not have any heart failure decompensations, and was able to lead an active life. LVEF remained above 50% and treatment for heart failure with improved LVEF was continued according to the guidelines [21,22]. Control of INR was better compared to previous visits, and he did not suffer any further ischemic events. Genetic screening of first-degree relatives was recommended but was not done due to financial reasons. The mother of the patient did not fulfill phenotypical criteria for HCM, but had left and right atrial dilation, likely due to the decades-long atrial fibrillation with no other apparent reason for the dilation.

## 3. Discussion

Left ventricular hypertrophy, although typically encountered daily in clinical practice, merits investigation, and should there be any suspicion of conditions with a specific etiology, proper diagnostic work-up is needed. There are several phenomena observed in our patient that are telling of the existence of HCM—the presence of left ventricular hypertrophy from an early age, the asymmetricity of the hypertrophy, the early onset of atrial fibrillation, the slow-flow phenomenon on coronary angiography, and the higher thromboembolic risk, as evidenced by an ischemic stroke at a relatively young age as well the presence of left atrial thrombosis despite the use of anticoagulants.

**Pathophysiological and clinical aspects:** Many particularities distinguish the hypertrophy in HCM compared to left ventricular hypertrophy that results from abnormal loading conditions or strenuous exercise. The hypertrophy that occurs in the latter two is an attempt to comply with the increased cardiac output demand. In the physiological adaptation, observed in athlete’s heart, there is a proportional increase in myocyte mass, extracellular matrix, and microvasculature of the heart, while in pathological conditions, such as aortic valvular disease—particularly aortic stenosis—and arterial hypertension, the increase in myocardial mass is not accompanied by an adequate proliferation of new vessels, and myocardial fibrosis appears over time, deterring the normal contractile and filling function [23]. In HCM, histologically, there are profound changes in terms of the forms of myocytes, the presence of binucleation or abnormal nuclei, a higher number of mitochondria, changes in the Golgi apparatus, and remodeling of the sarcomere T-tubule [12,13]. There is also a severe disruption of the normal architecture of the heart in the affected areas, the appearance of interstitial fibrosis, and intercellular connection abnormalities [13,24], leading alongside the hypertrophy to increased stiffness in the heart muscle, impeded filling in the left ventricle, ischemia, diastolic dysfunction, and in end-stage disease to systolic failure.

Arrhythmias affect about 40% of patients with HCM, with atrial fibrillation being the most common—34.1% [25]—and sudden cardiac death rate determined to be 0.43% in adults [26]. HCM predisposes to the appearance of atrial fibrillation through various mechanisms. On the molecular level, there is a disruption in normal electric signaling due to the disarray in the architecture of the myocytes, further aggravated by the gradual accumulation of fibrosis due to chronic ischemia and increased left ventricular strain, leading to elevated pressures and electric remodeling in the left atrium [27]. The role of pulmonary vein isolation in HCM and atrial fibrillation is still debated, but there are trials with promising efficiency results [28]. The persistence of atrial fibrillation leads to significant hemodynamic worsening of HCM due to the lack of active atrial filing, while high ventricular rates additionally shorten the diastole and deter the filling of the non-compliant left ventricle [29].

HCM is known to independently increase the risk of thromboembolic events, and the use of anticoagulants is indicated in any detected episode of atrial fibrillation regardless of the CHA2DS2-VASc score result [14,30]. According to the results of a vast meta-analysis of 33 studies, the prevalence of thromboembolism in HCM with detected atrial fibrillation was 27%, with left atrial size and age being predictors of its occurrence and associated thrombotic complications [31]. Other factors such as left ventricular outflow tract obstruction, significant mitral insufficiency, heart failure, and late gadolinium enhancement are also related to the risk of thromboembolism [32]. Genetically determined atrial myopathy is another hypothesis for the development of atrial fibrillation and left atrial thrombosis [33,34], which might be the case in our patient, given the family history of AF and the additional presence of the *SCN5A* gene mutation. While both VKAs and DOACs appear similarly effective in HCM with atrial fibrillation, DOACs are associated with better safety, according to observational studies [35].

The presence of myocardial ischemia in HCM is mostly due to the mismatch between the increased oxygen demand of the hypertrophied myocardium and the remodeling of the coronary arteries [36], while chronic ischemic events contribute to the fibrosis progression [37]. Small-vessel coronary artery disease appears to involve both tunica media and the intima and leads to severe narrowing [37]. Additionally, there is evidence that the index of microcirculatory resistance, which reflects of small-vessel ischemia and fibrosis, is associated with the prognosis of HCM [38]. The increased intracardiac pressure during diastole has been also implicated in the worsening of the coronary flow dynamics [36]. The CMR-detected subendocardial fibrosis and slow-flow phenomenon in our patient is likely due to the described microvascular and hemodynamic abnormalities. The slow-flow phenomenon has been reported in patients with HCM [39,40] and is known to cause myocardial scarring and result in CMR-detectable myocardial fibrosis [41].

**Imaging:** The most common ECG changes in HCM are the presence of voltage criteria for left ventricular hypertrophy, Q waves, and T wave abnormalities; yet, in a small number of patients, the ECG might be normal [42]. In our case, the ECG was not diagnostic for the condition, which could be due to several reasons, the most obvious of which is the high BMI of the patient (40.1 kg/m^2^), with excessive fat tissue attenuating the ECG signals [43]. Another likely explanation could be the presence of diffuse fibrosis, which is also known to reduce the amplitude of QRS complexes [44]. The right bundle branch block (RBBB) is known to further mask the outlook of the ECG by changing both repolarization and depolarization parameters, while the present RBBB itself is likely associated with the progressive fibrotic changes in HCM [45]. We assume that the lack of typical changes on ECG was partial to the delay of the diagnosis in our case.

The European Society of Cardiology defines the cut-off of 15 mm left ventricular wall thickness in any segment as diagnostic for the presence of HCM in adults, while hypertrophy of 13–14 mm might still be a sign of HCM [14]. Echocardiography, although a first-line imaging modality in cardiological practice, is not sensitive to the described complex structural changes that occur in the myocardium. CMR, while similarly non-invasive, provides a much better evaluation and differentiation from other conditions. The interpretation of the CMR findings in our case was challenging. The differential diagnosis included infiltrative cardiomyopathy (amyloidosis and Anderson–Fabry disease) and HCM with atypical phenotype. Clinically speaking, a cardiac form of amyloidosis, however, is very unlikely to remain asymptomatic for about two decades, and additionally, there was no systemic involvement or other amyloidosis stigmas such as valvular, right ventricular walls or atrial septal thickening, or apical sparing [46].

Anderson–Fabry disease was ruled out by enzyme testing. The described atypicality of the CMR finding might be in line with the specificity of the underlying genetic defects, as HCM is known for its heterogenic expression [47]. Phenotypically, the myocardial thickening was relatively mild and limited to the mid and partially basal septum. The ejection fractions of both ventricles were significantly reduced, a finding consistent rather with infiltrative cardiomyopathies and end-stage HCM. On the other hand, the ventricles were not dilated as one would expect in end-stage HCM. Restoration of LV ejection fraction with guideline-recommended treatment and normalization of thyroid function was documented at follow-up. The structural changes in the myocardium included a significant prolongation of the T1 and T2 relaxation time and extensive LGE, indicating enlargement of the interstitial space with increased water content and diffuse fibrosis or infiltration. The changes extended well beyond the area with increased myocardial thickness, involving, albeit to a different degree, all segments of the LV myocardium, far more than 15%, which might be related to higher sudden cardiac death risk [20], although is not yet considered a strict indication for ICD implantation. The diffuse presence of fibrosis even in non-hypertrophied regions is in fact in line with postmortem studies of HCM [48]. The pattern of the LGE varied throughout the myocardium and was mainly intramural (including in the thickened segments), but with some subendocardial distribution in the apical segments of the LV free wall and at the apex. This type of structural change is consistent rather with infiltrative cardiomyopathy though also reported in HCM with adverse remodeling subtype [49]. The presence of subendocardial LGE could be associated with microcirculatory dysfunction [13].

**Genetic aspects and prognostic implications:** The role of genetic counseling, genetic testing, and cascade screening in HCM is emphasized by the current guidelines of leading cardiovascular societies [14,15], as typically, the inheritance is autosomal dominant, and identification of pathogenic/likely pathogenic variants might allow for a better understanding of the prognosis of the disease, serve for prenatal planning, and determine the need for future follow-up of relatives. In our case, molecular genetic analysis made us more comfortable in establishing the diagnosis, when doubts were present from the performed imaging tests. A still unsolved problem is the relatively high price and the lack of health insurance reimbursement of such analyses, which unfortunately prevented us from performing cascade screening of the relatives of the patient. The *ACTC1* gene encodes the cardiac α-actin, the main component of the sarcomere thin filament. Interacting with myosin and Z-disk’s α-actinin or intercalating disks, actin has an essential role in both force generation and force transmission, and *ACTC1* gene mutations have been implicated in both hypertrophic and dilated cardiomyopathy [50,51,52]. Cardiac actin is a protein, composed of two domains, each constituted of two subdomains [53]. It has been proposed that *ACTC1* variants in the protein subdomain 1, which have a direct impact on myosin binding sites, may be classified as M-type mutations, including the variant p.His103Gln found in our patient [54]. Of note, M-class mutations have been found only in HCM patients to date. The p.Glu99Lys variant of the *ACTC1* gene has been shown to weaken the actomyosin interaction, thereby decreasing the force and motion generating properties of actin in a model expression system [55]. Study data show that myosin moves p.Glu99Lys thin filaments at significantly slower velocity compared to wild-type filaments, with an increase in ATP consumption and reduced efficacy, which may be associated with the development of HCM [56,57,58]. However, data on estimated myosin duty ratios using human M-class *ACTC1* variant proteins with myosin ATPase activity and in vitro motility data were not consistent (with only the p.Glu99Lys variant showing an increase in duty ratio), implicating the involvement of other mechanisms in the pathogenesis of the disease [59]. Additional research on p.Glu99Lys and other M-class cardiac actin variants showed increased calcium sensitivity in regulated thin filaments, which supports the hypothesis that HCM may be a result of the increased calcium sensitivity during myocardial contraction [60,61].

The variant p.His103Gln in the *ACTC1* gene has been previously reported in heterozygous state alone in one patient with HCM, included in a large cohort study, and in a combination with variant p.Lys994Arg in the *MYH7* gene in a 33-year-old female with a maximum left ventricular wall thickness of 21 mm and NYHA class I heart failure symptoms [62,63]. This evidence suggests that the p.His103Gln variant in the *ACTC1* gene is rare and more data and long-term observations are needed to better characterize the associated phenotype.

The *PLN* gene encodes phospholamban—a small 52-amino acid protein, existing both in a pentameric or monomeric form, which is expressed primarily in cardiac, smooth, and slow-twitch skeletal muscle tissue [64,65,66]. Phospholamban acts as a key regulator of cardiac sarcoplasmic reticulum Ca^2+^-ATPase (SERCA2a), a major enzyme in cardiac Ca^2+^ metabolism, involved in the transport of more than 70% of cytosolic Ca^2+^ into the sarcoplasmic reticulum [66].

The activity of SERCA2a is critical for the rate of Ca^2+^ transportation and amount of ions stored to be released during the next heartbeat; thus, SERCA2a’s function has a direct effect on heart relaxation and may influence contractility. The current understanding is that phospholamban constitutes of two domains—a hydrophilic domain (amino acids 1 to 30), including Ser^16^ and Thr^17^ sites for phosphorylation, which are essential for alleviating phospholamban’s inhibitory effect on SERCA2a in response to β-adrenergic stimulation, and a hydrophobic domain (amino acids 31 to 52), anchoring the protein to the sarcoplasmic reticulum membrane [65]. Data show that monomeric PLN directly interacts with SERCA2a, while the pentamer acts as a reservoir of monomeric PLN [66]. Null mutations in the *PLN* gene have been associated with lethal dilated cardiomyopathy in humans [67]. Study data from an explanted heart show that homozygosity for the p.Leu39Ter mutation in the *PLN* gene resulted in a reduction over 50% in *PLN* mRNA and no detectable PLN protein. The mutation expression models performed demonstrated absence of stable expression of phospholamban, misrouted localization to the cytosol or plasma membrane, and lack of inhibition of SERCA2a. Furthermore, data show that the p.Leu39Ter mutation prevents anchoring of PLN in the membrane, greatly reducing oligomerization and binding to SERCA2a [68].

The p.Leu39Ter mutation in the *PLN* gene was first described by Haghighi et al. [67] in two families with hereditary heart failure. In this study, a brother and a sister, carrying the p.Leu39Ter variant in the *PLN* gene in homozygous state, developed dilated cardiomyopathy and heart failure, requiring cardiac transplantation at ages 16 and 27, respectively. Interestingly, some of the heterozygous carriers of the variant in both families exhibited hypertrophy, while others developed dilated cardiomyopathy. One of the heterozygous individuals had no remarkable findings on echocardiography, showing incomplete penetrance of the disease phenotype. Heterozygosity for the p.Leu39Ter variant in the *PLN* gene has also been identified in two male patients with dilated cardiomyopathy [69,70]. The first one [69] was diagnosed at 42 years of age with an ejection fraction at admission of 32% and died at 43 years of age due to cardiogenic shock. The second patient [70] was diagnosed with dilated cardiomyopathy at the age of 40 years and presented with sustained ventricular tachycardia episodes and atrial fibrillation at 56 years of age. He died of end-stage heart failure at the age of 60.

Landstrom et al. [71] reported the p.Leu39Ter mutation in *PLN* in a 58-year-old male with HCM with a positive family history, diagnosed at 51 years of age, with septal and apical hypertrophy with a maximum left ventricular wall thickness of 24 mm, Wolff–Parkinson–White syndrome, left atrial enlargement, sinus bradycardia, conduction block, ventricular ectopy with symptomatic non-sustained ventricular tachycardia, and paroxysmal atrial fibrillation/flutter. The p.Leu39Ter variant in the *PLN* gene has also been found in a heterozygous state in a 61-year-old female patient with familial HCM and a clinical history of recurrent atrial fibrillation, palpitations, dyspnea, and presyncope and in large cohort studies of HCM patients [72,73,74]. A 14-year-old female cardiac arrest survivor was found to harbor the same nonsense variant in *PLN* in a heterozygous state, as well as a 57-year-old female with non-penetrant disease [75,76]. These observations suggest that the cardiovascular phenotype, associated with the p.Leu39Ter mutation in *PLN*, is highly variable, and is the most severe phenotype with the least favorable outcomes observed in homozygous mutation carriers ranging to a milder phenotype, showing incomplete penetrance in heterozygous carriers. The enhanced lusitropic and inotropic effects, resulting from PLN absence/dysfunction, might explain the observed DCM, complicated by heart failure, and HCM phenotypes in *PLN* mutation carriers. However, the influence of other poorly understood factors on clinical expression might not be ruled out. Of note, atrial fibrillation has been reported in two of the heterozygous mutation carriers with HCM and one with DCM, although this association needs to be further elucidated in future investigations.

The *SCN5A* gene encodes a 2016-amino acid cardiac sodium channel α-subunit protein with four homologous domains, consisting of six transmembrane α-helical segments each [77]. Opening of sodium channels, triggered by membrane depolarization, allows for the influx of Na^+^ from the extracellular space into the cytosol, followed by a rapid inactivation with closure of the open pore [78]. It has been established that the SCN5A C-terminal domain, spanning residues 1773 through 2016, is part of a molecular complex together with the protein III-IV loop, which is necessary to stabilize the channel closed gate and minimize channel reopening during membrane depolarization. Pathogenic mutations disrupting this interaction have been associated with a delay in cellular repolarization and a diverse clinical phenotype. The variant p.His1943Tyr in the *SCN5A* gene, identified in our patient, which is localized in the SCN5A C-terminal domain, has not been previously reported in ClinVar or the published literature, or identified in the control populations of the gnomAD v2.1.1 project, so there is currently no sufficient evidence to determine its pathogenicity.

Several study reports show that HCM patients, carrying multiple mutations in one or more genes, have younger age at diagnosis, greater maximum LV wall thicknesses and left atria size, severe disease progression, and a higher risk for adverse outcomes, including cardiovascular death, sudden cardiac death, and heart failure-related death, compared to patients, carrying single mutations [9,11,61,79]. Interestingly, none of the *PLN* p.Leu39Ter mutation carriers in the clinical cases to date, reported in detail, have been found to harbor a sarcomeric gene mutation as well. Taken together, this evidence indicates the indispensable role of expanded panel analysis in molecular genetic testing in patients with HCM and risk assessment of inheritance in affected families.

## 4. Conclusions

Contemporary management of patients with unexplained left ventricular hypertrophy should rely on multi-modality imaging and utilization of molecular genetic analysis, if necessary, especially given the fact that cardiomyopathies often affect people at active age. Efforts should be made to make these methods more accessible to patients in everyday clinical practice. Despite their higher initial cost, they can significantly reduce serious complications such as stroke and heart failure with proper diagnostic workup.

## Figures and Tables

**Table 1:** Timeline of events.

Time/Period/Age	Clinical Presentation	Management	Therapy
Early 20s	“Hypertrophy of the heart” noted during obligatory military service	No recommendations given. Explained with increased physical activity during military service.	
2016, age 41	Ischemic stroke	Risk factor: Arterial hypertension, grade I; Heart enlargement described on chest X-ray; No specific follow-up recommended.	Clopidogrel 75 mg Piracetam 2400 mg Irbesartan 75 mg
July 2020, age 44	A first episode of persistent atrial fibrillation (AF) registered	Anticoagulant treatment initiated and planned for transesophageal echocardiography (TEE) and electrical cardioversion (ECV).	Dabigatran 2 × 150 mg added
August 2020, age 44	TEE visualized thrombosis of the left atrial appendage (LAA)		Dabigatran switched to Acenocoumarol
September 2020, age 45	TEE negative for thrombosis of the LAA	Successful ECV	Acenocoumarol continued; Bisoprolol 5 mg as an anti-recurrence treatment
March 2021, age 45	First episode of decompensated heart failure, NYHA III-IV class; A new episode of persistent AF, duration longer than two months	Management of failure according to guidelines; TEE not performed due to INR not being in target; Asymmetric left ventricular hypertrophy and reduced ejection fraction noted; Further diagnostic work-up recommended—cardiac magnetic resonance (CMR) and genetic testing; Test for Anderson–Fabry disease—negative; CMR study positive for cardiomyopathy with diffuse fibrosis, possibly hypertrophic or infiltrative.	At admission: Amiodarone 400 mg; Torasemide 20 mg; Irbesartan 75 mg; Acenocoumarol; At discharge: Torasemide 50 mg; Spironolactone 50 mg; Acenocoumarol; Bisoprolol 5 mg; Sacubitril/valsartan 2 × 24/26 mg; Allopurinol 150 mg; Dapagliflozin 10 mg
April 2021	Compensated state of heart failure Ejection fraction improved to 45%	Selective coronary angiography—negative for epicardial vessel disease, but slow-flow phenomenon present in all coronary arteries TEE revealed LAA thrombosis while in INR therapeutic interval	
From 2021 and onwards	Compensated, greatly improved functional capacity Ejection fraction 50%	Refuses pulmonary vein isolation for atrial fibrillation	Torasemide reduced to 10 mg and then to 5 mg
March 2023		Molecular genetic testing revealed three variants, two of which consistent with hypertrophic cardiomyopathy and one possibly related to familial atrial fibrillation. Cascade screening recommended	

AF—atrial fibrillation; TEE—transesophageal echocardiography; ECV—electrical cardioversion; LAA—left atrial appendage; INR—international normalized ratio of prothrombin; CMR—cardiac magnetic resonance; NYHA—New York Heart Association.

**Table 2:** Echocardiographic findings.

LA A-P Diameter	LA Volume	Ind. LA A-P	LAVI	RA Diameters	RA Area	RA Volume	Ind. RA Volume
56 mm	130 mL	22.2 mm/m^2^	52 mL/m^2^	40 × 59 mm	24 cm^2^	65 mL	26 mL/m^2^
IVSd	PWLVd	IVSd/PWLVd	LV EDD	LV ESD	LV EDV	LV ESV	LVEF
16–17 mm;	8 mm;	2	48 mm	40 mm	163 mL	98 mL	35–40%
LV GLS	PSD	MV E-wave	e’sept.	e’lat.	E/e’ ratio	TAPSE	IVC
12.2%	57.2 ms	1.01 m/s	0.06 m/s	0.08 m/s	14.42	16 mm	20 mm, no collapse
RV basal diameter	RV EDA	RV ESA	RV FAC	RVFW	PA AT	TR Vmax	Kinetics
41 mm	21.4 cm^2^	14 cm^2^	35%	4 mm	148 ms	2.2 m/s	Diffuse hypokinesia

LA—left atrium; LA A-P diameter—anterior–posterior diameter of the LA; ind.—indexed to body surface area (BSA); LAVI—left atrium volume index; RA—right atrium; IVSd—thickness of the interventricular septum in diastole; LV—left ventricle; PWLVd—thickness of the posterior wall of the left ventricle; LV EDD—left ventricular end-diastolic dimension; LV ESD—left ventricular end-systolic dimension; LV EDV—left ventricular end-diastolic volume; LV ESV—left ventricular end-systolic volume; LVEF—left ventricular ejection fraction (Simpson’s biplane method of discs); LV GLS—left ventricular global longitudinal strain (speckle tracking-derived); PSD—peak systolic dispersion; MV E-wave—peak velocity of the early diastolic mitral valve inflow; e’sept—peak velocity of the septal e’ wave of the early diastolic filling; e’lat—peak velocity of the lateral e’ wave of the early diastolic filling; TAPSE—tricuspid annular plane systolic excursion; IVC—inferior vena cava; RV basal—right ventricular basal diameter; RV—right ventricle; RV EDA—end-diastolic area of right ventricle; RV ESA—end-systolic area of right ventricle; RV FAC—right ventricular fractional area change; RVFW—free wall of right ventricle; PA AT—pulmonary artery acceleration time and TR Vmax—maximum velocity of the tricuspid regurgitation jet.

## Data Availability

The original contributions presented in this study are included in the article; further inquiries can be directed to the corresponding authors; patient’s privacy restrictions apply.
